# Prevalence of Anti-Adeno-Associated Virus Immune Responses in International Cohorts of Healthy Donors

**DOI:** 10.1016/j.omtm.2019.05.014

**Published:** 2019-06-07

**Authors:** Anita Kruzik, Damir Fetahagic, Bettina Hartlieb, Sebastian Dorn, Herwig Koppensteiner, Frank M. Horling, Friedrich Scheiflinger, Birgit M. Reipert, Maurus de la Rosa

**Affiliations:** 1 Baxalta Innovations GmbH, a member of the Takeda group of companies, Vienna, Austria

**Keywords:** AAV, adeno-associated virus, gene therapy, anti-AAV prevalence, anti-AAV antibody response, anti-AAV T cell response, clinical assays, international cohorts

## Abstract

Preexisting immunity against adeno-associated virus (AAV) is a major challenge facing AAV gene therapy, resulting in the exclusion of patients from clinical trials. Accordingly, proper assessment of anti-AAV immunity is necessary for understanding clinical data and for product development. Previous studies on anti-AAV prevalence lack method standardization, rendering the assessment of prevalence difficult. Addressing this need, we used clinical assays that were validated according to guidelines for a comprehensive characterization of anti-AAV1, -AAV2, -AAV5, and -AAV8 immunity in large international cohorts of healthy donors and patients with hemophilia B. Here, we report a higher than expected average prevalence for anti-AAV8 (∼40%) and anti-AAV5 (∼30%) neutralizing antibodies (NAbs), which is supported by strongly correlating anti-AAV IgG antibody titers. A similar anti-AAV8 NAb prevalence was observed in hemophilia B patients. In addition, a high co-prevalence of NAbs against other serotypes makes switching to gene therapy using another serotype difficult. As anti-AAV T cell responses are believed to influence transduction, we characterized anti-AAV T cell responses using interleukin-2 (IL-2) and interferon-γ (IFN-γ) ELISpot assays, revealing a similar prevalence of IFN-γ responses (∼20%) against different serotypes that did not correlate with NAbs. These data, along with the long-term stability of NAbs, emphasize the need to develop strategies to circumvent anti-AAV immunity.

## Introduction

Recombinant adeno-associated virus 8 (AAV8) is a promising vector for gene therapy because it lacks pathogenicity in humans, provides long-term transgene expression with negligible integration into the host genome and, based on results in mice, is considered to have the best hepatocyte transduction efficiency among all AAV serotypes.^1^, ^2^, ^3^ AAV8 and AAV5 serotypes are currently favored for gene therapy, owing, among other reasons, to a potentially lower neutralizing antibody (NAb) prevalence compared with AAV2, which was widely used until clinical trials revealed that even low NAb titers against AAV2 can have an impact on the effectiveness of gene therapy.^4^ Nonetheless, preexisting immunity to AAV8 is still considered to be a major hurdle. Even low titers of NAbs against AAV8 have been shown to block *in vivo* transduction, and CD8 T cells specific for AAV8 are thought to kill transduced hepatocytes, preventing transgene expression.^4^ Consequently, patients with preexisting NAbs against AAV8 are currently ineligible for AAV8 gene therapy. To be able to dose patients efficiently and include all patients in need of therapy, it is necessary to reliably identify clinically relevant anti-AAV8 NAb titers and T cell responses.

Current studies suggest a wide prevalence range for NAbs against the different AAV serotypes, potentially due to analysis of different populations and the use of non-standardized assays with different sensitivities. The majority of reported anti-AAV8 NAb prevalences range between 20% and 30%, compared with 30% and 60% for AAV2, the serotype with the highest prevalence.^5^ However, even higher prevalences, up to 100% for AAV2 and up to 94% for AAV8, have been found.^6^, ^7^, ^8^, ^9^ AAV5 is often considered to be a serotype with very low NAb prevalence; reported prevalences range from 4% to 50%.^9^, ^10^, ^11^ Furthermore, longitudinal NAb titer stability needs additional investigation, as this has not yet been studied extensively in adults, the relevant patient group for current clinical trials.

No study has correlated anti-AAV8 NAbs with immunoglobulin (Ig) isotypes and IgG subclasses. To describe the characteristics of anti-AAV8 NAb responses and the potential involvement of T cell help, correlations need to be assessed in large cohorts from different regions using validated assays. The IgG subclass profile, however, has been analyzed, describing IgG1 as the predominant subclass, with only low levels of IgG2, -G3, and -G4 in all serotypes investigated, including AAV8.^10^, ^12^ This suggests the involvement of T cell help, because class switch is considered to be predominantly T cell dependent in antiviral immune responses.^13^, ^14^ Therefore, a correlation with AAV8-specific T cell responses would potentially support the presence of cytotoxic CD8 T cell responses. Although the preexisting prevalence of capsid-specific T cell responses has been assessed in other studies for AAV2, it has not been studied for AAV8.

The present international study used around 200 donors in different cohorts from the United States and Europe to map anti-AAV2, -AAV5, and -AAV8 immunity and correlate the antibody and cellular responses. To achieve the highest sensitivity in the *in vitro* NAb assay, reporter construct preparations were depleted of empty capsids. We report higher than expected NAb prevalence, which was supported by correlating IgG levels but did not correlate with anti-AAV T cell responses detectable in the circulation. To address the lack of assay standardization, we used validated clinical assays where the assay specifications and defined materials and controls were fully disclosed. These assays are currently used in clinical development. Moreover, the biological relevance of the NAb titers assessed by our *in vitro* NAb assay was investigated in more detail and has been published separately.^15^

## Results

### High Prevalence of Clinically Relevant Antibodies against AAV8

**Figure 1:**
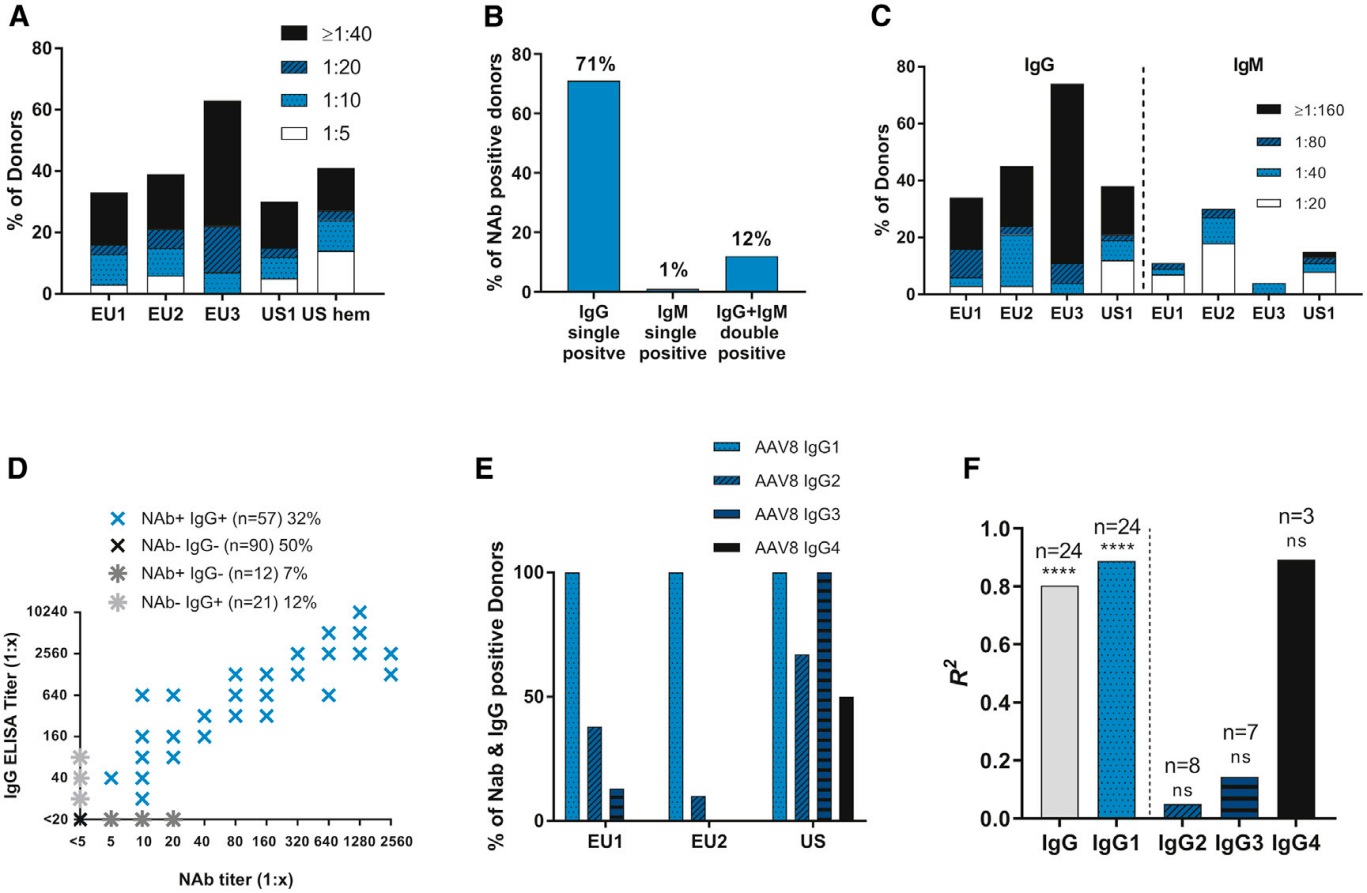
Antibody Response against AAV8 Prevalence and titers of NAbs, IgG, and IgM against AAV8 in cohorts of healthy donors and patients with hemophilia B from different geographical regions. (A) NAb prevalence (% of donors) against AAV8 in 180 healthy donors (EU1 cohort: n = 60, EU2 cohort: n = 33, EU3 cohort: n = 27, and US1 cohort: n = 60) and 29 patients with hemophilia B (US hem cohort). (B) The percentage of NAb-positive donors with IgG or IgM detected by ELISA with a starting dilution of 1:20. For 1% IgM single positive (one donor), IgG was detectable in the IgG ELISA with 1:5 as a starting dilution. (C) IgG and IgM against AAV8 in the 180 healthy donors (group and n as in A), indicating that switched IgG antibodies were predominant. (D) Correlation between NAbs and IgG against AAV8 (linear regression: n = 180, *R*^2^ = 0.8499, p < 0.0001). (E) Distribution of IgG subclasses. From 90 healthy donors, IgG subclasses were analyzed in all samples positive for NAb and IgG (EU1: n = 8, EU2: n = 10, and US1: n = 6). (F) Correlation between NAbs and IgG subclasses. Linear regression of NAb titers to IgG subclass titers was calculated; *R*^2^ values and significance of the correlations are depicted in the graph; ****p < 0.0001; ns: not significant.

Geographic differences in anti-AAV8 NAb prevalence were assessed in three cohorts of healthy donors from Europe, a cohort of healthy donors from the United States, and a cohort of patients with hemophilia B from the United States (Figure 1A). A high average anti-AAV8 NAb prevalence of 38% was found, varying from 32% (US healthy donor cohort) to 63% (Eastern Europe healthy donor cohort). The US hemophilia B cohort had an anti-AAV8 NAb prevalence of 41%, suggesting that the disease does not have an impact on anti-AAV8 NAb prevalence. Statistical analyses showed that the 63% prevalence reported in the Eastern European cohort was significantly higher compared with other cohorts; all other differences between cohorts were not significant (Kruskal-Wallis test). Age, sex, ethnic background, and the hemophilia B condition were not found to influence NAb titers.

**Figure 2:**
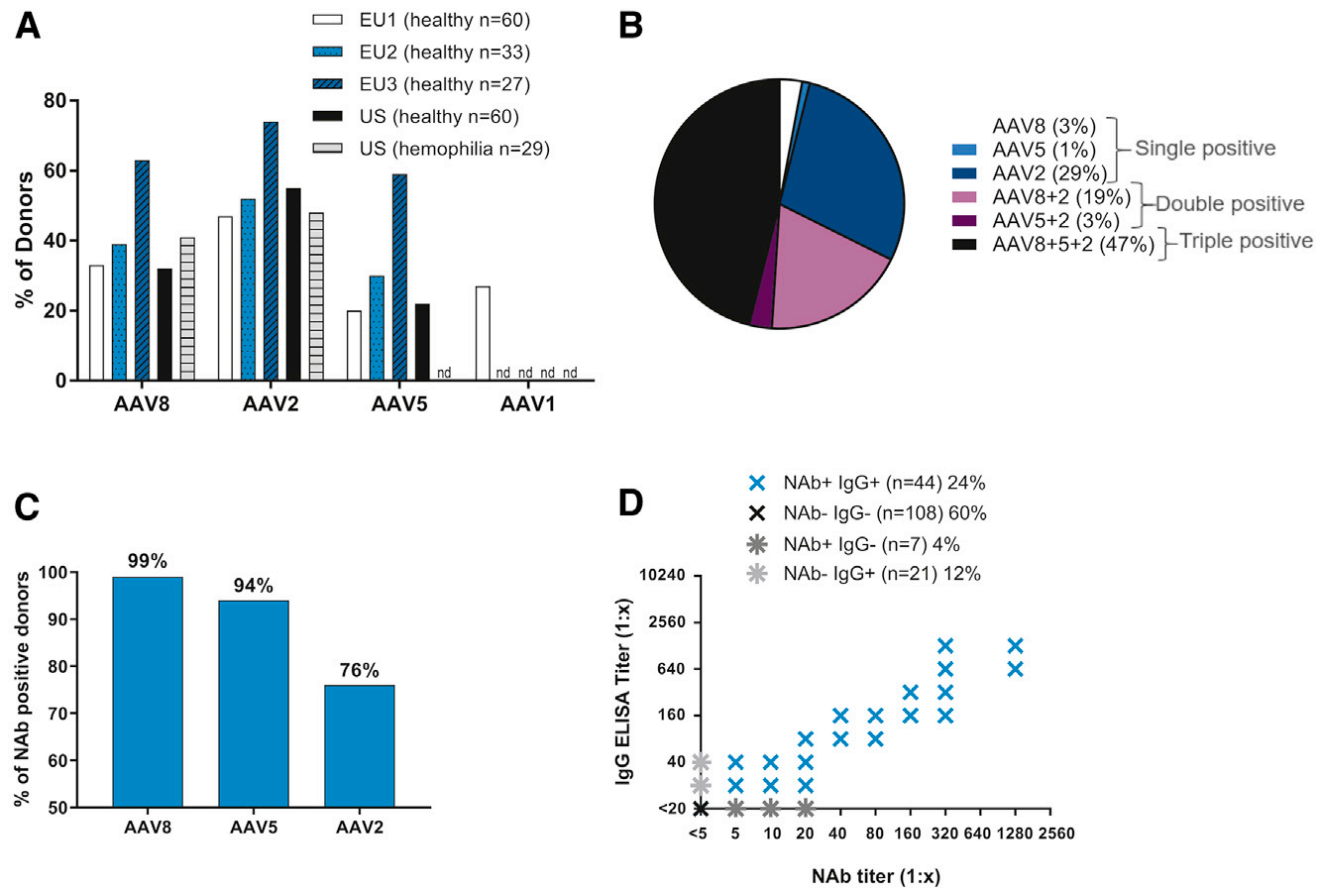
Co-prevalence of Antibody Responses against Different AAV Serotypes (A) Prevalence (% of donors) of NAbs against AAV8, AAV2, AAV5, and AAV1 in healthy donors and patients with hemophilia B from different regions. nd, not determined. (B) Co-prevalence (% of donors) of NAbs against AAV8, AAV2, and AAV5. The graph includes all donors from the EU1, EU2, EU3, and US2 cohorts from (A) that were positive for at least one of the serotypes. (C) The proportion of NAb-positive donors with IgG, suggesting that NAbs against AAV8 and AAV5 are always class switched. IgG ELISA was carried out with a starting dilution of 1:5. (D) Correlation between NAbs and IgG against AAV5 (linear regression: n = 180, *R*^2^ = 0.8652, p < 0.0001).

The high NAb prevalence is supported by a high prevalence of IgG against AAV8. Using the standard starting dilution of 1:20, 83% of the donors with anti-AAV8 NAbs showed anti-AAV8 IgG responses: 71% had IgG-only responses, and another 12% had IgG and IgM responses against AAV8 (Figure 1B). By modifying the ELISA (starting dilution, 1:5), 99% of NAb-positive donors had detectable IgG (Figure 2C), suggesting that IgG is responsible for AAV8 neutralization.

Overall, the total prevalence of IgG against AAV8 was found to be 35%, 45%, and 74% among the three cohorts from Europe, and 37% in the cohort from the United States (Figure 1C). IgM specific to AAV8, which was co-expressed with IgG, was found in only 10%, 30%, and 4% of all donors in the three cohorts from Europe and 15% in the cohort from the United States (Figure 1C). Accordingly, NAb titers showed a high correlation with IgG (Figure 1D) but not with IgM (Figure S1B), indicating that a T cell-dependent class switch potentially occurs in 99% of individuals and may be needed for NAb development.

To investigate whether an IgG subclass predominantly correlates with the neutralizing phenotype, we analyzed IgG subclasses in two smaller cohorts from Europe and one from the United States (Figure 1E). IgG1 was found in all donors positive for NAb and IgG, whereas the other subclasses were much less prevalent (Figure 1E) and had lower titers (Table S1), reflecting the typical IgG subclass pattern of a viral infection. In the small number of samples from the United States (n = 6), more IgG2, -G3, and -G4 were found than in the European cohorts (Figure 1E). Correlation of the IgG subclass titers with NAb titers further revealed that IgG1 correlated most with the neutralizing phenotype (Figure 1F), whereas IgG2, -G3, and -G4 showed no significant correlation with NAb titers (Figure 1F).

### The Assessment of NAb Prevalence for Other AAV Serotypes Reveals a High Co-prevalence

To assess co-prevalence to other AAV serotypes, we analyzed NAbs against AAV1, AAV2, and AAV5. Across the different cohorts, AAV2 NAbs were more prevalent (47%–74%) than AAV8 NAbs (prevalence: 32%–63%), whereas AAV5 and AAV1 NAbs were found to be less prevalent (20%–59% and 27%, respectively; Figure 2A). In addition, only about one-third of NAb-positive donors were positive for NAbs against only one serotype, whereas the other two-thirds were positive for NAbs against two or three serotypes (Figure 2B). Donors with NAbs against only one serotype had lower NAb titers than those with NAbs against more than one serotype (p < 0.0001). In 87% of donors, those with NAbs against multiple serotypes had higher NAb titers against AAV2, suggesting that lower titers against AAV5 and AAV8 in the same sample may be due to the cross-reactivity of anti-AAV2 NAbs.

Analysis of IgG against AAV5 revealed that, similar to AAV8, donors with NAbs against AAV5 had IgG in most cases (94%; Figure 2C), supporting a higher prevalence of anti-AAV5 than initially reported in all cohorts.^10^ By contrast, 24% of donors with NAbs against AAV2 had no detectable IgG (Figure 2C), but did have IgM against AAV2 (data not shown), suggesting that IgM antibodies play a role in neutralizing AAV2. Accordingly, AAV5 NAb titers showed a good correlation with IgG (Figure 2D), whereas AAV2 NAb titers correlated only to a lesser extent with IgG (Figure S2B).

### AAV8-, -5-, and -2-Specific T Cell Responses Have a Similar Prevalence

**Figure 3:**
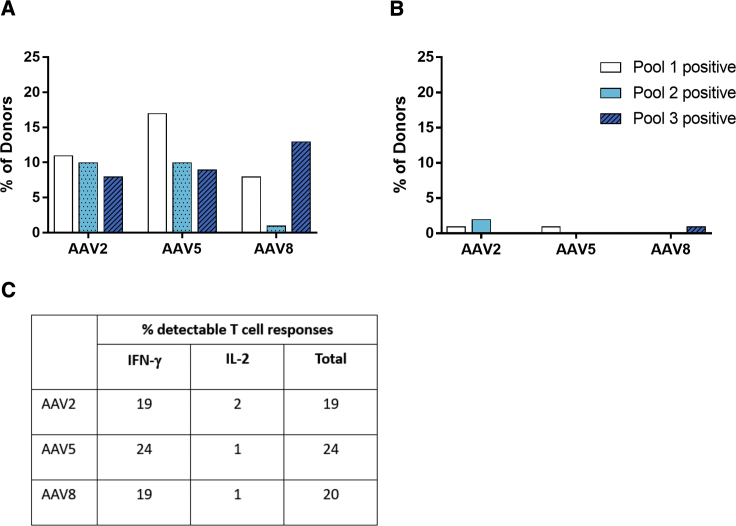
Prevalence of T Cell Responses against Different AAV Serotypes Frequency of capsid-specific (A) IFN-γ- or (B) IL-2-secreting T cells detected using ELISpot analysis in PBMCs from 90 healthy donors. PBMCs were stimulated with AAV8, AAV5, or AAV2 peptide pools for 18 to 24 h. The percentage of donors positive for each peptide pool is depicted, showing a similar prevalence for all serotypes. (C) Table showing percentage of donors with detectable IFN-γ and IL-2 T cell responses against AAV2, AAV5, and AAV8.

As anti-AAV T cell responses are thought to potentially eliminate transduced hepatocytes, their prevalence was assessed with a sensitive interferon (IFN)-γ enzyme-linked immunospot (ELISpot) assay in peripheral blood mononuclear cells (PBMCs) from 90 healthy donors from Europe and the United States. An interleukin-2 (IL-2)-specific ELISpot assay was also used to detect potentially AAV-specific naive or central memory T cells. Overall, 19% of the donors had detectable IFN-γ-secreting cells in the circulation (Figures 3A and 3C). Of these positive donors, 29% were positive for peptide pool 1, 16% for pool 2, and 53% for pool 3. A further 12% were positive for pools 1 and 3 (data not shown). There was no statistically significant difference between healthy donors and patients with hemophilia B. Circulating IFN-γ-secreting T cells specific for AAV5 or AAV2 were found in 24% and 19% of the donors, respectively (Figures 3A and 3C). By contrast, IL-2-producing T cells were found to be rare with all serotypes tested (Figures 3B and 3C). In summary, we found an equally robust T cell response against the different serotypes.

### AAV-Specific Antibody Responses Do Not Correlate with T Cell Responses

**Figure 4:**
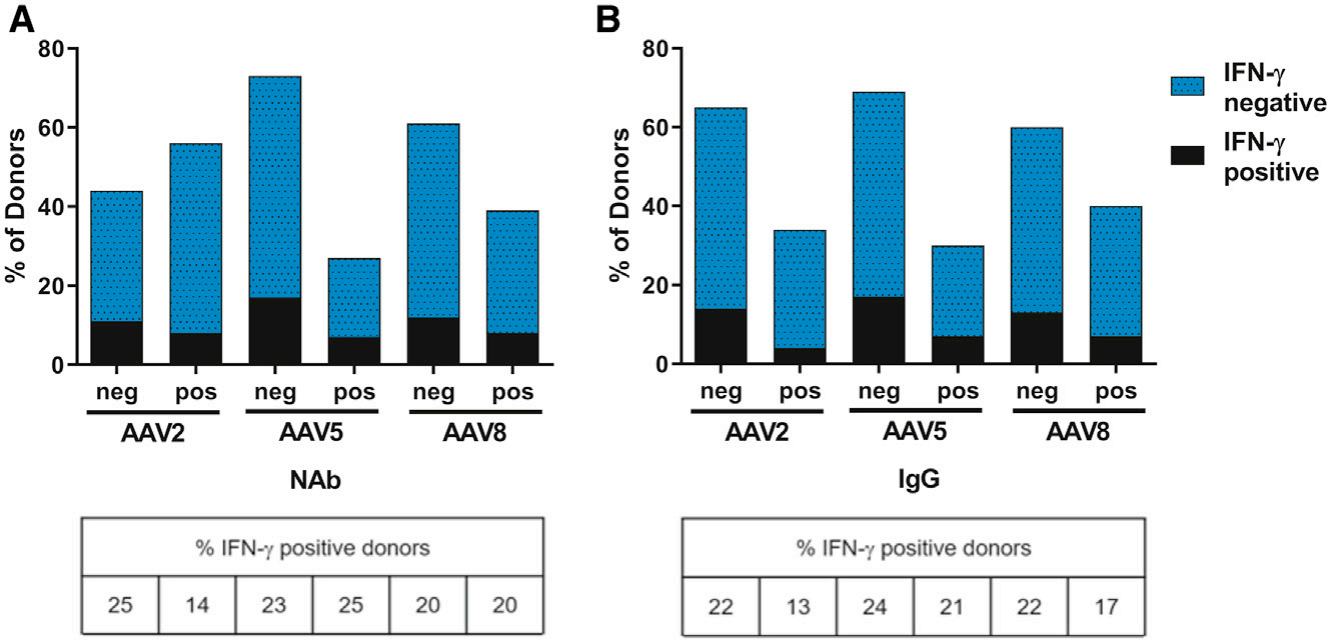
Correlation between T Cell and Antibody Responses against Different AAV Serotypes AAV-specific T cells were analyzed using an IFN-γ ELISpot assay in PBMCs from 90 healthy donors (the same donors as in Figure 3). The prevalence of T cells directed against AAV8, AAV5, and AAV2 was compared between (A) NAb-positive and NAb-negative or (B) IgG-positive and IgG-negative donors, indicating no correlation between T cell and antibody responses against AAV. The tables depict the proportion of IFN-γ-positive donors from each column of the figure.

Figures 1D and 2D show a good correlation between NAb and anti-AAV IgG responses; however, no correlation between antibody responses and anti-AAV T cell responses were found (Figures 4A and 4B). For AAV8, we found that 20% of the NAb-negative donors had detectable IFN-γ-secreting cells, as did 20% of the NAb-positive donors (Figure 4A). Moreover, in statistical analyses, the NAb titer level did not significantly correlate with anti-AAV T cell responses, although 99% of NAb-positive donors were positive for IgG (Figure 2C), which potentially indicates the presence of AAV8-specific T cells. Similarly, we found that 25% and 23% of the donors negative for NAbs against AAV2 and AAV5, respectively, had detectable IFN-γ-secreting cells, compared with 14% and 25% of the NAb-positive donors, respectively (Figure 4A). The prevalence of IL-2-secreting anti-AAV T cells was too low to allow for correlation analysis with antibody responses. As T cell responses are a prerequisite for highly efficient NAb titers and longer-lasting immune responses, the major histocompatibility (MHC) haplotypes of the 90 healthy donors used for the ELISpot assay were sequenced and compared with the T cell responses. We did not identify any predominant MHC I alleles responsible for the recognition of AAV8, AAV2, or AAV5 by CD8 T cells. Similarly, no MHC II haplotypes predominantly responsible for the recognition of AAV8, AAV2, or AAV5 by CD4 T cells were found.

### Longitudinal Stability of NAb Titers Is High

**Figure 5:**
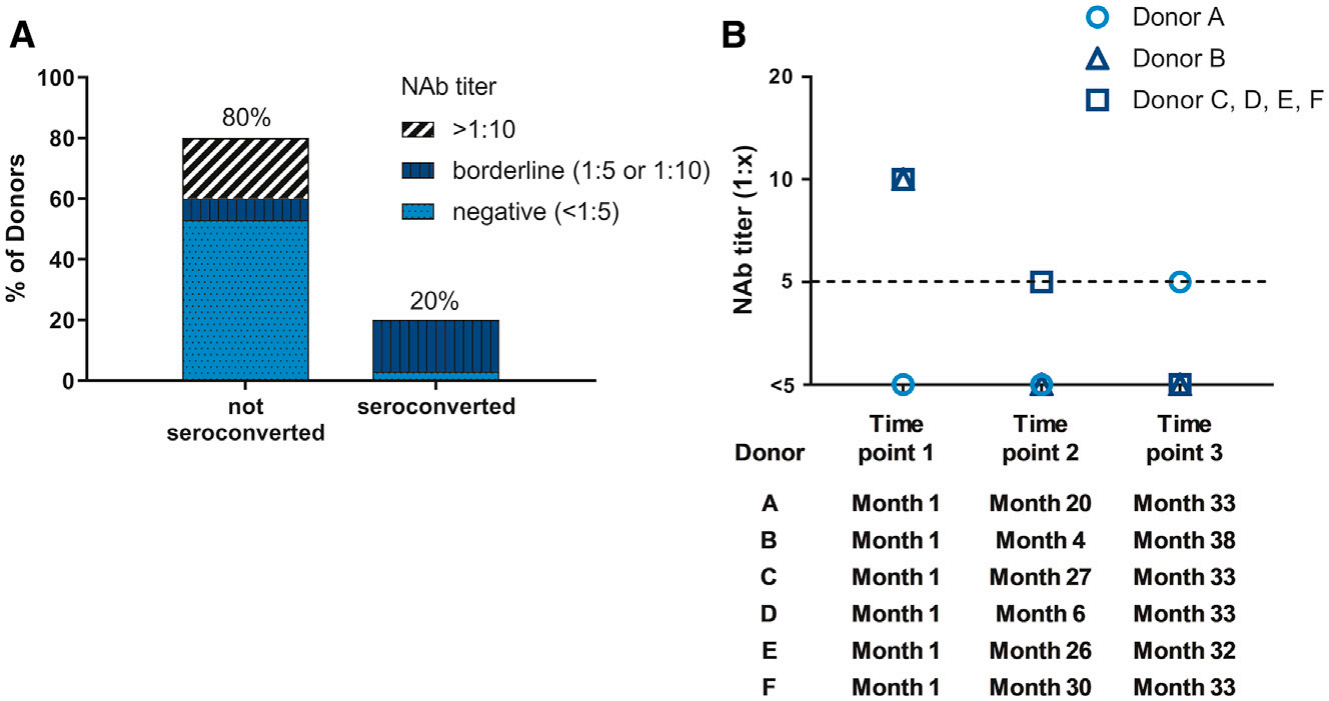
Development of Anti-AAV8 NAb Titers over Time (A) NAb titers from 30 healthy donors were analyzed at least three times during the course of up to 3 years. The figure shows the serological status of donors at the first time point. Within the donors that did not seroconvert (n = 24), 16 donors were negative, 2 donors had borderline NAb titers, and 6 had NAb titers >1:10 at the first time point. In contrast, none of the donors that seroconverted (n = 6) had an NAb titer >1:10. One of these donors was negative and 5 had borderline NAb titers at the first time point. (B) Fluctuations in NAb titer of the donors who seroconverted over time. The dashed line shows the seroconversion threshold (assay cutoff).

To assess the stability of anti-AAV NAbs induced by wild-type AAV infections, we used a representative cohort of 30 healthy donors to analyze anti-AAV8 NAbs over the course of nearly 3 years. In total, 80% of the donors did not seroconvert and were stable regarding positive-negative evaluation (Figure 5A). Only 13% (n = 4) of these donors had fluctuations exceeding assay variation, and 51% (n = 16) were anti-AAV8 NAb negative and stable within ±1 titer step. In total, 20% (n = 6) of the donors seroconverted during the 3 years. All of these donors had borderline NAb titers of <1:5, 1:5, or 1:10, and 83% (n = 5) had changed from positive to negative status. Only one negative donor (6%) changed from negative to positive status (titer, 1:5) (Figure 5B). Overall, the data suggest a relatively high stability of NAb titers and indicate that borderline samples have to be considered carefully because of potential titer fluctuations. Similarly, NAb titers against AAV8 remained constant for up to 3 years in patients with hemophilia B after gene therapy (Figure S3).

## Discussion

Preexisting immunity against the vector is a major challenge in AAV gene therapy. Among other reasons, AAV8 gene therapy has been developed to treat a larger proportion of patients because it has lower NAb prevalence than that of AAV2.^4^ However, we found a profoundly high prevalence of NAbs against AAV8 in healthy donors. Regional variations indicate that between 32% and about 65% of patients would be ineligible for AAV8 gene therapy. A similar distribution and high prevalence can be expected in clinical studies, because no major difference was detected between the hemophilia B cohort and healthy donors. Therefore, anti-AAV8 NAbs would be expected to block access to AAV8 gene therapy for a significant proportion of patients.

Low NAb titers of 1:5 blocked transgene expression in a mouse model for AAV8 gene therapy (Figure S4), showing the relevance of low NAb titers and, thus, of the prevalence reported here. Our results are further supported by a confirmatory study in mice, involving the same reagents, and by the demonstration of the biological relevance of the *in vitro* assay (and its 1:5 cutoff).^15^ Moreover, the higher anti-AAV8 NAb prevalence (compared with previous reports) was supported by the determination of anti-AAV8 antibody isotypes and IgG subclasses, using a different assay format, and by the high correlation between IgG and anti-AAV8 NAb titers. The use of reporter constructs containing 90% full capsids in the NAb assay ensured high assay sensitivity and minimized the impact of empty capsids. Therefore, we consider the higher prevalence reported here to be clinically relevant. Neutralization correlated with IgG but not IgM, indicating that class switch, which is usually T cell dependent in antiviral immune responses, is common during anti-AAV8 NAb development. Accordingly, none of the samples with IgM but without detectable IgG were neutralizing, suggesting that the AAV immune response is predominantly T cell driven. T cell-driven antiviral antibody responses are considered to be long lasting. Hence, the anti-AAV8 NAb titers remained constant over 3 years in adult healthy donors and patients with hemophilia B, consistent with recent findings in patients with Duchenne muscular dystrophy.^12^ However, borderline (≤1:10) anti-AAV8 NAb titers may change over time, because the 20% of donors who seroconverted were “borderline donors” (25% of all borderline donors). This change of titers has to be considered with caution because it is within the assay variance of ±1 titer step.

In summary, although the anti-AAV8 NAb prevalence was high, it was lower than the prevalence of anti-AAV2 NAbs (54%). Our data do not support the presence of neutralizing factors other than antibodies, as suggested previously;^10^ in our study, only 12 of 180 samples had NAbs without detectable IgG. This may have been caused by different assay starting dilutions (NAb assay, 1:5; IgG ELISA, 1:20). Indeed, 11 of these 12 donors had detectable IgG in a modified ELISA (Figure S1A; starting dilution, 1:5), indicating that unknown factors account for NAb activity only in rare cases.

The prevalence of anti-AAV5 NAbs is strongly debated because serotypes with lower NAb prevalence are being sought, to allow treatment access for more patients. Although the prevalence of NAbs against AAV5 (28%) is lower than for AAV8 and AAV2, a substantial number of patients would still be excluded from clinical trials because of preexisting NAbs. High correlation with anti-AAV5 IgG indicates that this high anti-AAV5 NAb prevalence is accurate and clinically relevant. Importantly, these data show that anti-AAV5 immune responses are of similar quality, which may indicate that AAV5 has an immunogenic character similar to that of AAV8. Moreover, in common with AAV8, the data indicate the potentially T cell-dependent character of the anti-AAV5 immune response.

Furthermore, owing to the high co-prevalence of anti-AAV NAbs, the potential for simply switching to another wild-type serotype is limited. Overall, 50 of 51 donors with anti-AAV5 NAbs also had anti-AAV2 NAbs. Of these donors, 46 also had anti-AAV8 NAbs, and only these triple-positive donors had NAb titers of ≥1:20 against AAV5 or AAV8. Although the assays have, not identical, but similar sensitivities, this suggests cross-reactivity. Similar conclusions can be drawn from previous studies.^10^, ^16^ The sequence homology of AAV5 compared with AAV2 and AAV8 is 61.2% and 61.1%, respectively, whereas that of AAV2 and AAV8 is 83.6%.^17^ However, separate infections cannot be excluded, because AAV5 and a virus from the same clade as AAV8 have been isolated from humans.^17^, ^18^

Viral infections are considered to elicit NAbs, CD4 T cell responses, and cytotoxic CD8 responses at the same time as part of the crucial host defense mechanism.^19^ IgG data suggest a potential T cell dependency and the presence of anti-AAV CD4 and CD8 responses. However, the variability of the anti-AAV2 T cell prevalence in previous studies is considerable (50%;^20^ 4%^21^), highlighting the need for standardized assays. Our validated ELISpot showed the anti-AAV prevalence to be approximately 20% for all serotypes for IFN-γ, again suggesting a similar quality of T cell responses against the different AAV serotypes. We also used an IL-2 ELISpot to detect AAV-specific memory T cell subsets^20^, ^22^ that do not produce IFN-γ. However, IL-2 was barely detectable, implying that IL-2-producing memory cells are very rare. Interestingly, although anti-AAV T cells have been described to be cross-reactive between AAV serotypes,^23^ hardly any triple-positive samples (with T cells against AAV2, AAV5, and AAV8) were identified.

Despite the T cell dependency suggested by IgG subclasses, no correlation between anti-AAV T cell responses and anti-AAV NAb titers were found. Similar observations have been reported for AAV1.^24^ Therefore, it could be speculated that anti-AAV T cells are tissue resident^25^ or circulating at very low frequencies.^21^ In addition to IFN-γ, the major cytokine in antiviral immune response,^26^ IL-2 responses, a marker for activation of naive cells and central memory cells,^27^ were also investigated. However, by applying this additional readout, no correlation with anti-AAV8-antibody responses was detected. TNF-α was included in an additional cohort of 30 donors, suggesting that, in contrast to a recent study,^28^ TNF-α does not correlate with NAbs (data not shown). However, we believe that in these two cases the cohort size was too small. This topic requires further investigation.

The presence of anti-AAV8 T cells in the absence of anti-AAV8 antibodies may be explained in 44% of the donors by the co-prevalence of potentially cross-reactive antibodies against other AAV serotypes. The remaining 56% of anti-AAV T cell responses without detectable anti-AAV antibodies cannot be explained by antibodies against the serotypes we analyzed, but could be explained by cross-reactive antibodies against other AAV serotypes. The ELISpot assay also does not seem to be too sensitive, as it does not appear to show false-positive results in a clinical gene therapy trial (ClinicalTrials.gov: NCT01687608). Although anti-AAV responses seem to be generally T cell dependent, we could not find a correlation between MHC I and II haplotypes, probably owing to the restricted sample size.

In summary, on account of non-correlating anti-AAV8 T cell and NAb responses, we have shown that a statistically significant proportion of patients (32%–63%) would need to be excluded from AAV gene therapy, owing to anti-AAV8 immunity. As a result of the relatively high prevalence of NAbs to AAV8 and other AAVs, only the development of tailored strategies to circumvent or overcome the anti-AAV immune response^15^ will allow the use of AAV gene therapy in this group of patients.

## Materials and Methods

### Healthy Donors and Patients with Hemophilia B

Samples were taken from cohorts of healthy donors from Europe (obtained from in.vent Diagnostica GmbH [EU2 cohort n = 33, EU3 cohort n = 27, http://www.inventdiagnostica.de] or collected in Austria [EU1 cohort n = 60, as approved by the ethics committee of the Medical University, Vienna]) and the United States (US1 cohort n = 60, StemCell Technologies, https://www.stemcell.com). All human participants gave written informed consent. In addition, a cohort of 29 patients with hemophilia B from the United States was included. Most of the donors were Caucasian and their ages ranged from 19 to 84 years. The hemophilia B cohort and one cohort from Europe (EU3 cohort) consisted of male donors only, whereas the other cohorts included 50% female donors. The United States cohort comprised 50% Caucasian and 30% Hispanic donors, with the remainder distributed among African American, Asian, Native American, and Middle Eastern donors.

### NAb Assay

To analyze NAbs, HuH7 cells (hepatocarcinoma cell line) were seeded in 96-well plates (2 × 10^4^ cells per well). After 20 to 24 h, the cells were infected with adenovirus 5 (helper virus) to improve transduction efficiency. Highly purified AAV1-, AAV2-, AAV5-, or AAV8-luciferase reporter constructs (90% full capsids) were incubated with plasma or serum samples in 2-fold serial dilutions (1:5 starting dilution) for 1 hour. For AAV1, AAV5, and AAV8, the cells were then infected with the sample and reporter construct mixture. The MOI was titered during assay development and was found to be 3 × 10^4^. For AAV2, MOI 3 × 10^2^ was optimal. After 20 to 24 h, the cells were washed and lysed (One-Glo Luciferase Assay System, https://www.promega.com) and luminescence was measured. The last dilution of a sample that reduced luciferase expression by at least 50% represented the NAb titer. The NAb assay was validated according to regulatory requirements for clinical studies^29^ (assay variation, ±1 titer step).

### ELISA

Anti-AAV binding antibodies were detected by ELISA. AAV2, AAV5, or AAV8 capsids were coated on 96-well plates overnight at 4°C. Plates were blocked with 1% casein in phosphate-buffered saline (PBS) for 1 h, then incubated with plasma or serum samples diluted in 1% casein in PBS (1:20 starting dilution, then 2-fold serial dilutions) for 2 h. For antibody detection, horseradish peroxidase-conjugated secondary antibodies, specific to human IgG, -M, or -G subclasses, were incubated for 1 h, then tetramethylbenzidine was used as a substrate. Optical density was measured and samples were evaluated using a cutoff established specifically for each isotype and subclass. The ELISA was validated according to regulatory requirements for clinical studies.^29^ During validation, a cutoff was determined as the 95th percentile of negative and false-positive samples based on screening and confirmatory ELISA carried out in 144 donors. Cutoff assessment was performed separately for each of the ELISAs (AAV8 IgG, AAV8 IgM, AAV8 IgG1, AAV8 IgG2, AAV8 IgG3, AAV8 IgG4, AAV2 IgG, and AAV5 IgG2). In some cases, a 1:5 starting dilution was used.

### ELISpot

PBMCs were isolated from sodium-citrated whole blood, collected, and isolated by centrifugation for 30 minutes (1,600 *g*, 20°C), and the plasma supernatant was removed. The PBMCs were washed twice with PBS and then frozen at 1 × 10^7^ cells/mL at −150°C. ELISpot analysis was carried out without pre-stimulation using CTL ELISpot kits according to the manufacturer’s protocol (Cellular Technology Limited; http://www.immunospot.com). Dilutions of 4 × 10^5^ PBMCs/well were stimulated with AAV8, AAV2, or AAV5 peptide pools (15-mer, offset 5; pool 1, peptides 1–50; pool 2, peptides 51–100; and pool 3, the remaining peptides). As established during assay validation, samples were evaluated as being positive when they had at least 24 spots, and the spot count was more than three times higher than the unstimulated negative control. The ELISpot and cell processing protocols were validated according to regulatory requirements for clinical studies.

### MHC Sequencing

High-resolution MHC sequencing was performed using the Ion Torrent/PGM platform, as described by Rothberg et al.^30^ The genes were amplified using whole blood as a source of DNA, then fragmented and analyzed by next-generation sequencing with the Ion Torrent system.

### Statistical Analysis

Statistical analyses were performed with GraphPad Prism Software, Version 7.03 (https://www.graphpad.com). The Mann-Whitney test was used to determine the significance between two groups, and the Kruskal-Wallis test with Dunn’s *post hoc* test was used for more than 2 groups. The Pearson correlation coefficient was calculated to analyze correlations. Values of p ≤ 0.05 were considered significant.

All analyses were considered to be exploratory, and therefore no adjustment for multiplicity was applied. The objective of the analysis was to derive further hypotheses to be investigated.

### AAV Vectors

Purified AAV1-, AAV2-, AAV5-, or AAV8-luciferase reporter constructs and AAV8-Factor IX vectors were used (University of North Carolina Gene Therapy Vector Core, Chapel Hill, NC, USA).

### Data-Sharing Statement

Data are the property of Baxalta Innovations GmbH, a member of the Takeda group of companies, Vienna, Austria. Access can be requested by contacting Takeda’s legal representative.

## Author Contributions

A.K. designed the research, established the validated assay formats, performed the experiments, analyzed and interpreted the data, and wrote the manuscript. D.F. performed the experiments. B.H. and S.D. established the validated assay formats and analyzed and interpreted the data. H.K. analyzed and interpreted the data. F.M.H., F.S., and B.M.R interpreted the data and facilitated the study. M.d.l.R. designed the study, analyzed and interpreted data, and wrote the manuscript. All authors reviewed the manuscript critically for intellectual content and participated in drafting and/or revising the manuscript. All authors approved the final version for submission.

## Conflicts of Interest

All authors were employees of Baxalta Innovations GmbH, a member of the Takeda group of companies, Vienna, Austria at the time of the current analysis. A.K., B.H., and M.d.l.R. are employees of Baxalta Innovations GmbH, a member of the Takeda group of companies, Vienna, Austria. D.F. and S.D. were employees of Baxalta Innovations GmbH, at the time of the current analysis, and are now employees of Hookipa Biotech GmbH. H.K. was an employee of Baxalta Innovations GmbH, at the time of the current analysis, and is now an employee of Pfizer. F.M.H. was an employee of Baxalta Innovations GmbH, a member of the Takeda group of companies, at the time of the current analysis, and is now an employee of IMC University of Applied Sciences Krems. F.S. and B.M.R. are employees of and stockholders in Baxalta Innovations GmbH, a member of the Takeda group of companies, Vienna, Austria.

## Supplemental Information

Document S1. Table S1, Figures S1–S4, and Supplemental Materials and Methods

Document S2. Article plus Supplemental Information
