# Supplementary material for: Prevalence of Anti-Adeno-Associated Virus Immune Responses in International Cohorts of Healthy Donors
Source: Mol Ther Methods Clin Dev. 2019 Jun 7;14:126–33. doi: 10.1016/j.omtm.2019.05.014 (PMC6629972; doi:10.1016/j.omtm.2019.05.014)
Supplement: Document S1. Table S1, Figures S1–S4, and Supplemental Materials and Methods [file mmc1.pdf]

OMTM, Volume *14*

Supplemental Information

Prevalence of Anti-Adeno-Associated Virus Immune Responses in International Cohorts of Healthy Donors

Anita Kruzik, Damir Fetahagic, Bettina Hartlieb, Sebastian Dorn, Herwig Koppensteiner, Frank M. Horling, Friedrich Scheiflinger, Birgit M. Reipert, and Maurus de la Rosa

Table S1. NAb titers compared with IgG and IgG subclass titers. IgG subclasses were analyzed in all samples positive for IgG from 90 healthy donors.

**NAb-positive donors**							
Donor	NAb	IgG	IgG1	IgG2	IgG3	IgG4	IgM
B029	1:2560	1:2560	1:2560	1:40	<1:20	<1:20	<1:20
V015	1:2560	1:2560	1:5120	<1:20	1:160	<1:20	<1:20
V088	1:2560	1:2560	1:2560	<1:20	<1:20	<1:20	<1:20
V111	1:2560	1:1280	1:2560	<1:20	<1:20	<1:20	<1:20
U011	1:1280	1:10240	1:10240	1:320	1:320	1:40	<1:20
B027	1:640	1:640	1:1280	<1:20	<1:20	<1:20	<1:20
B011	1:320	1:1280	1:1280	<1:20	<1:20	<1:20	<1:20
U021	1:320	1:1280	1:1280	1:40	1:20	1:20	1:20
B012	1:160	1:640	1:640	<1:20	<1:20	<1:20	<1:20
B013	1:160	1:640	1:640	<1:20	<1:20	<1:20	1:20
U005	1:160	1:640	1:1280	1:20	1:80	<1:20	1:320
U006	1:160	1:640	1:640	<1:20	1:20	<1:20	<1:20
U030	1:160	1:640	1:1280	1:20	1:80	1:20	<1:20
V005	1:160	1:320	1:1280	<1:20	<1:20	<1:20	<1:20
B022	1:80	1:640	1:640	<1:20	<1:20	<1:20	1:20
B009	1:20	1:160	1:160	<1:20	<1:20	<1:20	<1:20
B020	1:20	1:80	1:80	<1:20	<1:20	<1:20	<1:20
V110	1:20	1:80	1:80	<1:20	<1:20	<1:20	<1:20
V073	1:20	1:80	1:80	1:40	<1:20	<1:20	<1:20
V095	1:10	1:640	1:20	1:640	<1:20	<1:20	<1:20
V037	1:10	1:80	1:40	1:80	<1:20	<1:20	1:20
U024	1:10	1:40	1:40	<1:20	1:80	<1:20	<1:20
B023	1:10	1:40	1:20	<1:20	<1:20	<1:20	<1:20
B025	1:5	1:40	1:40	<1:20	<1:20	<1:20	1:40

Figure S1. Correlation between NAbs and IgG against AAV8. (A) Correlation between NAbs and IgG was calculated using the results of the validated, clinical IgG ELISA with a starting dilution of 1:20, and using a modified version of the ELISA starting with a 1:5 dilution. Linear regression was calculated; R^2 values are depicted in the graph. **** significant correlation between NAb and IgG. (B) No correlation between NAbs and IgM against AAV8 (linear regression: n = 180, R^2 = .0003961, P = .7909).

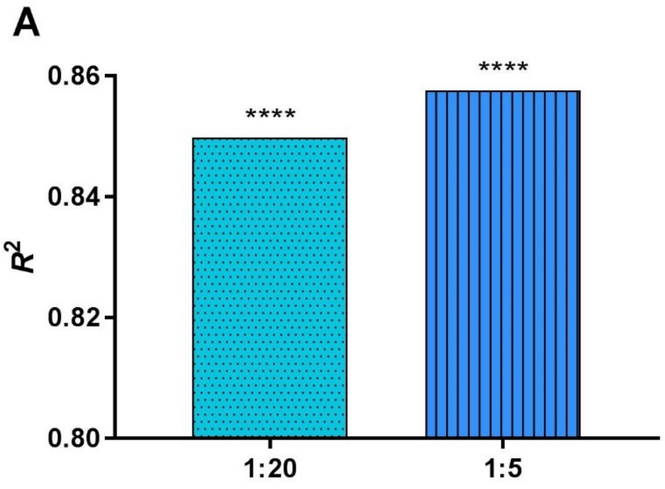

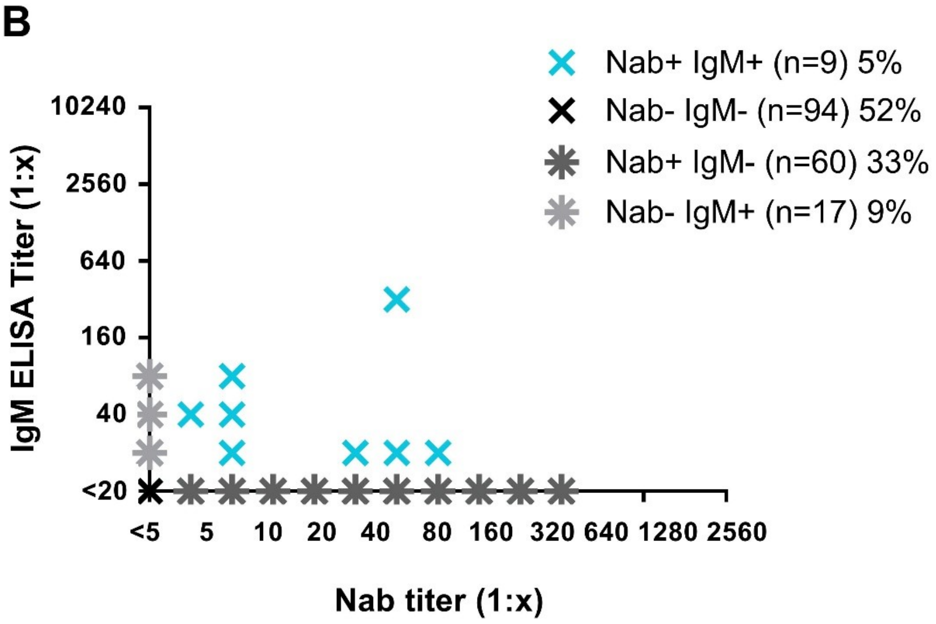

Figure S2. Co-prevalence of antibody responses against different AAV serotypes. (A) Prevalence of IgG against AAV8, AAV2, and AAV5 in healthy donors from different regions. (B) Correlation between NAbs and IgG against AAV2 (linear regression: n = 180, R^2 = 0.7291, P < .0001).

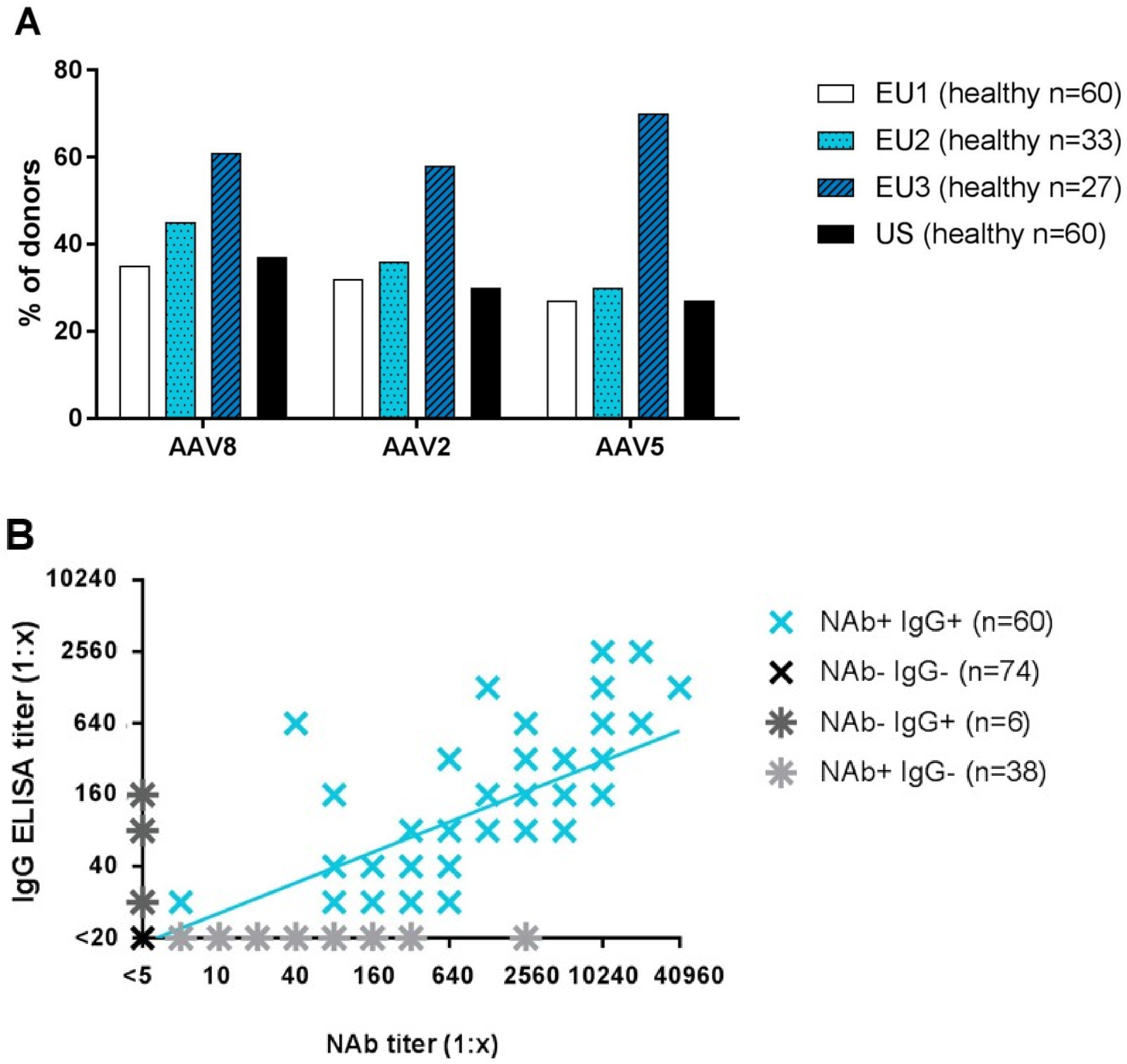

Figure S3. Development of anti-AAV8 NAb titers over time in patients with hemophilia B. NAb titers from 8 patients with hemophilia B were analyzed during the course of 12 to 24 months.

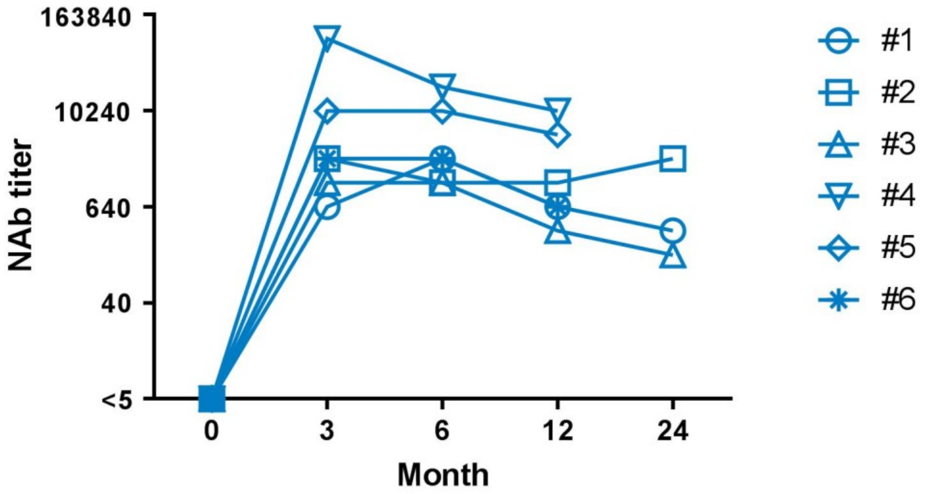

Figure S4. Biological relevance of low anti-AAV8 NAb titers. An anti-AAV8 NAb titer of 1:5 was established in mice by passive transfer of 4 different human plasma samples containing NAbs. Subsequent AAV8-Factor IX (FIX) gene therapy was completely blocked by this low titer of NAbs. For each human plasma sample, AAV8-FIX gene therapy in the absence of NAbs was carried out in parallel in a control group. Group size was 5 mice.

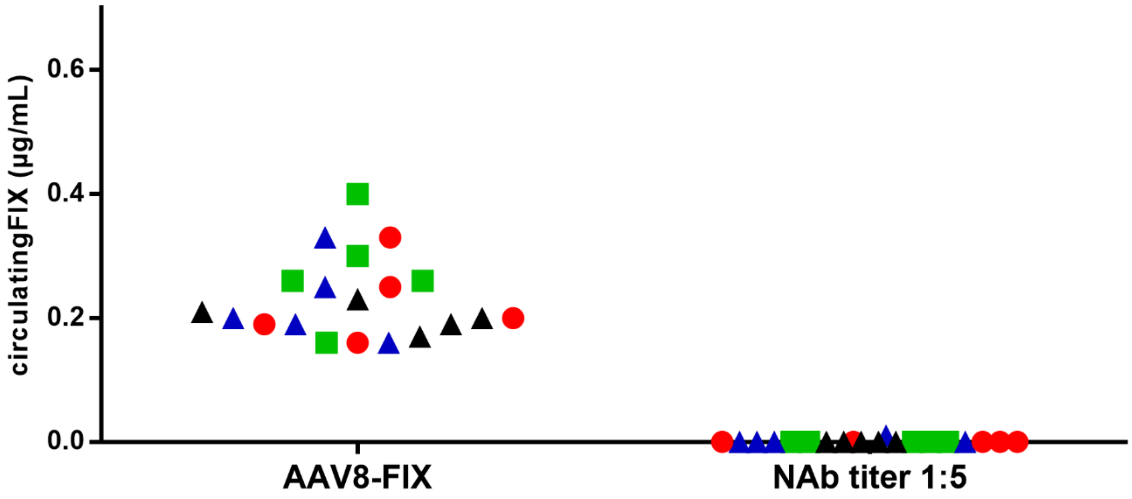

Supplemental Method

In vivo model for gene therapy

50 µl of human plasma containing anti-AAV8 NAbs were injected intravenously to NOD/SCID (NOD.CB17-Prkdcscid/J, Charles River, France) mice to establish a NAb titer of 1:5 in the mouse circulation. After 3 hours, blood was collected to confirm the NAb titer by the NAb assay. At the same time, the mice received 150 µL AAV8 FIX (2 $\times 10^{10}$ vg/mouse). After 14 days, blood was collected, and FIX expression was analyzed by ELISA. In brief, anti-human FIX IgG was coated to 96-well plates, plates were blocked, and serial dilutions of samples or standards were loaded. FIX was detected by a peroxidase-labeled anti-human FIX secondary antibody.
